# Comparison of the effects of Duphaston and Cetrotide on oocyte and embryo quality in women undergoing ICSI: A cross-sectional study

**DOI:** 10.18502/ijrm.v13i11.7965

**Published:** 2020-11-22

**Authors:** Niloofar Motaref, Sheyda Jouhari, Afsaneh Mohammadzadeh, Somaieh Kazemnejad, Narges Madadi, Sadaf Eghtedari, Abolfazl Ghoodjani

**Affiliations:** Reproductive Biotechnology Research Center, Avicenna Research Institute, ACECR, Tehran, Iran.

**Keywords:** Duphaston, Cetrorelix, Dydrogesterone, COH, GnRh antagonis.

## Abstract

**Background:**

Premature luteinizing hormone (LH) surge is one of the causes for assisted reproductive technology cycle cancellation, and it is needed to find novel approaches with improved efficacy and safety profile.

**Objective:**

To compare the effects of Duphaston and Cetrotide on the prevention of premature LH surge and characteristics of retrieved follicles and embryos in women undergoing intracytoplasmic sperm injection.

**Materials and Methods:**

In this retrospective cross-sectional study, 200 patients who were administrated recombinant follicle-stimulating hormone from the third day of menstruation cycle were included. When the follicular diameter reached above 13-14 mm, Cetrotide was prescribed in the control group, while in the case group, Duphaston was taken orally from the third day of cycle. The retrieved oocytes were fertilized in vitro by intracytoplasmic sperm. The level of hormones on the third day of menstruation and the characteristic of follicles, oocytes, and embryos were compared between the two groups.

**Results:**

Duphaston successfully inhibits premature LH surge. There was no significant difference in the level of follicle-stimulating hormone, estradiol, and LH between the case and control groups (p > 0.05). However, results also showed that Duphaston causes more oocyte retrieval in comparison with Cetrotide (p = 0.04). Although, the number of follicles above 14 mm, mature oocyte, and the total number of viable embryos in the case group was slightly higher, it did not reach a significant difference compared with the control group (p > 0.05).

**Conclusion:**

Duphaston could be used as an appropriate medication instead of gonadotropin-releasing hormone antagonists in women undergoing controlled ovarian hyperstimulation. Duphaston prescription not only prevents premature LH surge but also improves the number of retrieved oocytes.

## 1. Introduction

About 8-12% of reproductive-aged couples suffer from infertility worldwide (1). Over the last decade, assisted reproductive technology (ART) have drawn considerable attention to overcome the problem of infertility (2). Interestingly, the premature luteinizing hormone (LH) surge is one of the main causes for ART cycle cancellation. In IVF cases an early surge of LH can compromise the oocyte resumption (3). In recent years, several investigations have been done to reduce the incidence of early LH surge by using gonadotropin-releasing hormone (GnRH) agonist and antagonists (4).

Adjuvant therapy with GnRH agonists could prevent premature LH surge. However, this may decrease the response to stimulation. Therefore, higher dose of the drug and long treatment period would be essential to reach suitable follicular development (5). In fact, the need for repeated measurements of serum LH, higher medication costs, risk of ovarian hyperstimulation syndrome due to use of higher dose of drugs are the main disadvantage of GnRH agonist protocols (6). Therefore, it is necessary to find novel approaches with improved efficacy, safety profile, and user convenience.

The initial investigations show that steroidal products such as oral contraceptive pills and synthetic progesterone may be an inexpensive and effective method of preventing LH and follicle-stimulating hormone (FSH) secretion (7). Furthermore, it is known that natural and synthetic progesterone suppress pulsatile GnRH and LH secretion (8). These effects propose that progesterone can be used as an alternative to a GnRH analogue for suppressing premature LH surge in controlled ovarian hyperstimulation (COH). One of the synthetic forms of progesterone is Duphaston (Dydrogesterone), whose molecular structure and pharmacologic effects are closely related to endogenous progesterone (9). The clinical efﬁciency of Duphaston in progesterone primed ovarian stimulation (PPOS) regimen were evaluated by Eftekhar and colleagues (10), who showed that Duphaston as an FSH adjuvant during the ovarian stimulation did not lead to similar mature oocyte retrieval.

The present study was conducted to compare the efficacy of Duphaston with Cetrotide in normally ovulating women undergoing COH in terms of hormone profiles, number of retrieved oocyte, and embryo quality.

## 2. Materials and Methods

### Study setting, patients, ovarian stimulation, and oocyte retrieval

This retrospective cross-sectional study was conducted at the Avicenna Infertility Clinic of Tehran and included. Women undergoing ICSI regimens for infertility treatment between September 2017 and December 2018. The FSH and estradiol levels of patients were assessed on the third day of menstruation in serum, following which they were divided into case and control groups.

Infertile women aged 20-40 yr, having an antral follicle count of at least 4 on the third day of menstrual cycle, FSH < 12 IU/L, absence of uterine anomalies and hydrosalpinx, and undergoing IVF first or second time were deemed eligible for the study. Whereas, the exclusion criteria of the study included: evidence of ovarian failure (lack of antral follicle count in sonography evaluation on the third day of menstruation), grade 3 or 4 endometriosis, every contraindication for ovarian stimulation, and systemic illness such as kidney failure.

A total of 200 patients were assessed for eligibility (each group 100 patients). Both groups were administered 150–225 IU recombinant FSH (rFSH-Gonal-f Merck Serono, Germany) from the third day of the menstruation cycle. Follicles were monitored through transvaginal sonography, 5–6 days after the gonadotropin stimulation. When the follicular diameter reached above 13–14 mm, Cetrotide (0.25 mg/d; Merck Serono, Germany) was prescribed in the control group, while in the case group, Duphaston (20 mg/d; Abbott Healthcare, America) was taken orally from the third day of cycle until the trigger day. From the time when three dominant follicles reached > 17 mm, 10,000 IU of hCG (Pooyesh Daroo, Iran) was prescribed for the final maturation of oocytes and ovulation. Oocyte retrieval was performed 34–36 hour after the trigger, and the retrieved oocytes were fertilized in vitro by ICSI.

### Hormone measurement

The level of LH, FSH, estradiol, and progesterone in the serum were measured by chemiluminescent method on the third day of menstruation. A lack of ovulation before the puncture day was considered as a lack of premature LH surge.

### Outcome 

The number of follicles above 14 mm, number of oocytes retrieved, their maturation step (Germinal vesicles, MI and MII), viable embryos and their grade (A, AB, B, and C) were assessed as the primary outcome and compared between the two groups. In this assessment, the embryo was evaluated according to the Cummins criteria on the third day of fertilization by number, regularity of blastomeres, and embryonic fragmentation grade. The demographic and clinical characteristics such as age, height, infertility duration, and the number of IVFs were also collected.

### Ethical consideration

This study was approved by the ethics committee of the Avicenna Infertility Clinic (IR.ACECR.avicenna.REC.1397.004). The research- ers guaranteed that participants' information remained anonymous and confidential during the study period and adhered to the principles of the Helsinki Declaration and the Ethics Committee of the Avicenna Infertility Clinic. All participants provided informed consent after counseling for infertility treatments and routine IVF procedures.

### Statistical analysis 

Data are presented as the Mean ± SD. Independent *t* test was applied to analyze the differences in the outcome of treatment between the two study groups. All analyses were performed using the Statistical Package for the Social Sciences, version 16.0, SPSS Inc, Chicago, Illinois, USA (SPSS). P-value < 5% was considered as statistically significant.

## 3. Results

### Patient characteristics

This study included 200 patients who were assigned to either the case group (Duphaston) or the control group (Cetrotide). The average age of the control group was 33 ± 4.1 yr and the case group was 32 ± 4.5 yr, and there were no significant differences between them (p = 0.26). The mean BMI in the case and control groups were 24.61 ± 3.32 and 24.49 ± 3.85, respectively, which shows no significant alterations between the two groups (p = 0.37). Further, data analysis also revealed that there was no significant differences between groups with respect to the duration of infertility (3.87 ± 3.85 vs 3.18 ± 3.31 year, p = 0.18). The mean number of IVFs in the case and control groups were 1.29 ± 0.45 and 1.62 ± 0.48, respectively, which showed that there were no significant differences between them (p = 0.21).

### Ovarian stimulation, follicles, oocyte, and embryo grades

The mean stimulation duration and the dose of rFSH and hCG were similar between the two groups. Table I compares the number of follicles above 14 mm, retrieved oocyte, mature (MII) and immature oocytes (GV and MI), viable embryos and their grade (A, AB, B, and C) between the two groups. Values for immature oocyte (MI), embryo grade AB, and number of retrieved oocytes were significantly different between the Duphaston (case) and Cetrotide (control) groups. Also, the number of follicles above 14 mm, mature oocyte (MII), total number of viable embryos, embryo grade B and C in the case group was slightly higher but did not reach a significant difference compared with the control group. The number of GV oocyte and embryos grade A in the control group was greater than the case group, however, these differences were not statistically significant.

### Hormone profile 

Table II presents the serum concentrations of FSH, estradiol, and LH on the third day of menstruation. As seen, on the third day of the menstrual cycles, there were no significant differences in level of FSH, estradiol, and LH between the case and control groups. Neither the case nor the control group had an oocyte ovulation before the puncture day. Therefore, it can be claimed that Duphaston prevents premature LH surge.

**Table 1:** Stimulation and embryonic characteristics of the case and control group


**Characteristic**	**Case (Duphaston)**	**Control (Cetrotide)**	P-value*
**Follicles above 14 mm**	9.6 ± 0.56	8.76 ± 0.51	0.25
**Retrieved oocytes**	13.35 ± 0.85	11.61 ± 0.76	0.04
**Immature oocyte (MI)**	1.11 ± 0.15	0.51 ± 0.10	< 0.001
**Mature oocyte (MII)**	11.01 ± 0.56	9.88 ± 0.63	0.20
**Immature oocyte (GV)**	0.7 ± 0.18	0.9 ± 0.22	0.51
**Viable embryos**	8.25 ± 0.56	7.34 ± 0.49	0.29
**Embryos A**	4.45 ± 0.37	4.89 ± 0.43	0.46
**Embryos AB**	2.08 ± 0.22	1.58 ± 0.18	0.01
**Embryos B**	0.77 ± 0.16	0.61 ± 0.11	0.43
**Embryos C**	0.40 ± 0.92	0.28 ± 0.06	0.31
Data presented as Mean ± SD. *Independent *t* test			

**Table 2:** Hormonal level in both groups on the third day of menstruation day


**Group**	**FSH**	**Estradiol**	**LH**
<statement> <title>Case </title> </statement>	6.73 ± 0.20	46.26 ± 1.93	6.12 ± 0.48
**Control**	7.25 ± 0.22	45.94 ± 2.68	6.16 ± 0.66
P-value*	0.11	0.90	0.95
Data presented as Mean ± SE. *Independent *t* test FSH: Follicular stimulating hormone; LH: Luteinizing hormone			

## 4. Discussion

This retrospective cross-sectional study revealed that in IVF/ICSI cases, oral prescription of Duphaston can be successfully used as an alternative to GnRH antagonist in patients undergoing controlled ovarian stimulation. Our findings confirmed that an oral delivery of Duphaston is an effective method in producing competent oocytes and blocking premature LH surges. Duphaston is a congener of progesterone with some but not all of the effects of progesterone, and it is safe without androgenic effects even at a high dose (11). Structural comparison of progesterone and Duphaston shows that Duphaston contains an extra double bond between 6- and 7-carbons. Furthermore, 10-carbon of Duphaston contain methyl group in α position, while methyl group on the 10-carbon of progesterone is located in β position (12, 13). These differences in the structure enhance the stability and bioavailability of Duphaston which improves its absorption and efficiency (14). This study demonstrated that Duphaston stimulates ovulation in a better manner and causes more oocyte retrieval in comparison with Cetrotide. Furthermore, in the case group who were administered Duphaston, the number of follicles above 14 mm, mature oocyte (MII), and the total number of viable embryos was higher than the control group; however, these differences were not significant. These results indicated that Duphaston was effective on controlled ovarian stimulation without any complications. These results are in agreement with previous studies (15, 16).

With regard to the progesterone level and oocyte/follicles quality, Zavareh and coworker (17) reported that increase of blood progesterone concentration may inhibit development of follicles and oocytes which lead to reduced fertility potential. Our finding demonstrated that Duphaston at the used dose in this study not only had no adverse effect on the development of follicles and oocytes but also caused more oocyte retrieval in comparison with the GnRH antagonists. These results are in agreement with the study of Hamdi and colleagues (18), who showed that medroxyprogesterone (a form of progesterone) has no adverse effects on oocyte or follicles development. It should be mentioned that further investigation is required to assess the higher dose of Duphaston or other progesterone congeners on follicles development. Study also revealed that no premature LH surge was observed during COH in patients and it seems Duphaston had suitable effects on the premature LH surge control. Additionally, in accordance with this study, Zhu and co-workers (9) reported no premature LH surge in patients who used Duphaston for COH. “The correlation between progesterone and LH surge is not completely clear. During COH, multiple follicles grow along with the dramatically increased estradiol levels, and the coordinated roles of estrogen and progesterone may be more powerful for suppressing LH levels" (3).

Richter and co-authors reported that LH surge takes place following the activation, transmission, and the GnRH surge (8, 19). Progesterone prevents premature LH surge by blocking the GnRH surge induction system (3). In agreement with this, it has shown that progestin administration during the normal follicular phase results in decrease of plasma levels of LH (20). Studies show that estradiol-induced LH surge-generating signal can be blocked by progesterone in early stages (immediately after estradiol removal) of signal transmission (8, 9, 21). Therefore, the role of progesterone in the LH surge depends on the time of its administration (17). Results indicated that to prevent premature LH surge in IVF cases, Duphaston could be used as an appropriate medication instead of Cetrotide in the patients who underwent controlled ovarian stimulation, because it had no complication in comparison to Cetrotide and is also more effective. Duphaston might be a good option in clinical practice, since oral administration is more patient-friendly than the injectable form.

The choice for either should be based mainly on the availability, cost, and side effects. The lack of pregnancy and neonatal outcomes are the limitations of this study. Therefore, complete additional studies are needed to provide more evidence about the efficacy of the Duphaston protocol and to illuminate its impact on pregnancy and children born from this novel regimen.

## 5. Conclusion

Duphaston can be used as an appropriate medication as an alternative to the GnRH antagonists. Duphaston prescription not only prevents premature LH surge but also improves the number of retrieved follicles and their quality. Oral administration of Duphaston provides easy access and better control over LH levels in comparison with the GnRH antagonists. Following the additional investigation about optimum dose and long-term safety of Duphaston, it could be used as a new regimen for a controlled ovarian stimulation in freeze-thaw cycles.

##  Conflict of Interest

The authors declare that they have no competing interests.
